# Relationship between Ocular Surface Alterations and Concentrations of Aerial Particulate Matter

**DOI:** 10.18502/jovr.v14i4.5441

**Published:** 2019-10-24

**Authors:** María A Gutiérrez, Daniela Giuliani, Atilio A Porta, Darío Andrinolo

**Affiliations:** ^1^University Extension Environmental Programme (PAEU), Faculty of Exact Sciences, National University of La Plata, Buenos Aires, Argentina; ^2^Center for Environmental Research (CIM), UNLP - CONICET, Buenos Aires, Argentina

**Keywords:** Environmental, Ocular Surface, Particulate Matter, Schirmer I Test, Vital Staining

## Abstract

**Purpose:**

To evaluate ocular surface alterations in two populations at different exposure levels to particulate matter (PM) in their living and work environments.

**Methods:**

A cross-sectional study was conducted, including 78 volunteers from Argentina who lived and worked under different pollution levels in an urban (U; *n* = 44) or industrial zone (I; *n* = 34). Mean exposure level to PM was evaluated. Responses to the Ocular Symptom Disease Index and McMonnies questionnaire were obtained from all subjects. Subsequently, an assessment through the Schirmer I test (ST), slit lamp microscopy, vital staining, and tear breakup time was conducted. Statistical analyses with Chi-square and Bartlett's tests, as well as Student's *t*-tests and principal component analysis (PCA), were performed.

**Results:**

Particles of size < 2.5 μm (PM${}_{2.5}$) level was significantly higher in the I group than the U group (*P* = 0.04). Ocular surface parameters including bulbar redness, eyelid redness, and the degree of vital staining with fluorescein (SF) and lissamine green (SLG) exhibited difference between the groups. With regards to the tear film, statistically significant differences in the ST value and meibomian gland dysfunction between the groups were detected (*P* = 0.003 and* P* = 0.02, respectively). Conjunctival SF and SLG, and ST values were identified as factors which could distinguish groups exposed to different PM levels.

**Conclusion:**

Subjects exposed to higher levels of PM in the outdoor air presented greater ocular surface alterations. Thus, ST, SF, and SLG values could be used as convenient indicators of adverse health effects due to exposure to air pollution.

##  INTRODUCTION 

The ocular surface comprises various structures in contact with the environment, namely the palpebral and bulbar conjunctival epithelium, corneoscleral limbus, corneal epithelium, and tear film, which provide anatomical, physiological, and immunological

This is an open access journal, and articles are distributed under the terms of the protection. The adnexal structures, including the anterior lamellae of the eyelids, eyelashes, meibomian glands, and lacrimal system, are essential for appropriate protection and function of the ocular surface.^[1]^ The ocular surface functions in generating good visual quality, nourishing and lubricating tissues, and protecting the eye against cellular debris and foreign particles.^[2]^ It can be affected by trauma, infections, or environmental factors, which may compromise the structural integrity of its components. This can lead to various forms of corneal and conjunctival dysfunction such as increasing order of severity, pain and itching,^[3]^ mild corneal abrasion to severe loss of stem cells, decreased vision,^[4]^ and blindness.^[5]^

Ocular surface disorders occur in patients with various conditions including limbal stem-cell deficiency and ocular surface disease (OSD) due to systemic diseases.^[1]^ Dry eye is an ocular surface disorder which has a prevalence rate of 5 to 50% worldwide. Dry eye is considered a multifactorial OSD characterized by a loss of homeostasis of the tear film, ocular symptoms of instability and hyperosmolarity of the tear film, and inflammation. Ocular surface damage and sensorineural anomalies may have etiological roles in this disease.^[6]^ Clinically, dry eye is characterized by a loss of tear volume, rapid breakup of the tear film, and increased evaporation of tears from the ocular surface.^[7]^

Recent studies have demonstrated subclinical alterations of the ocular surface that can be attributed to air pollution. These include changes in the tear break-up time (TBUT) and Schirmer I test (ST) value, ^[8,9,10,11,12]^ the incidence of palpebral affectations such as blepharitis,^[13]^ and effects on the ocular mucosa that indicate a significant positive association between exposure to the air pollutant nitrogen dioxide and goblet cell hyperplasia in the human conjunctiva.^[14]^ Additionally, reports have indicated that the presence of high concentrations of air pollutants such as nitric oxide, nitrogen dioxide, or sulfur dioxide makes the tear film increasingly acidic. Collectively, these findings suggest that symptoms of ocular discomfort and alterations in the TBUT could be used as bioindicators of the adverse health effects of air pollution due to vehicular traffic.^[10]^

The mechanisms by which air pollutants interact with the tear film, cornea, and conjunctiva remain unclear, although increases in MUC5AC mRNA level upon chronic exposure to particulate matter (PM) and nitrogen dioxide have been implicated.^[12]^ Further studies focused on the compensatory mechanisms of the ocular surface to changes induced by chronic exposure to air pollution and patient susceptibility are required to enable early treatment that prevents chronic disorders and promotes eye health.

This study aimed to evaluate ocular surface alterations in two populations at different exposure levels to PM in their living and work environments.

##  METHODS

###  Study Population 

**Figure 1:**
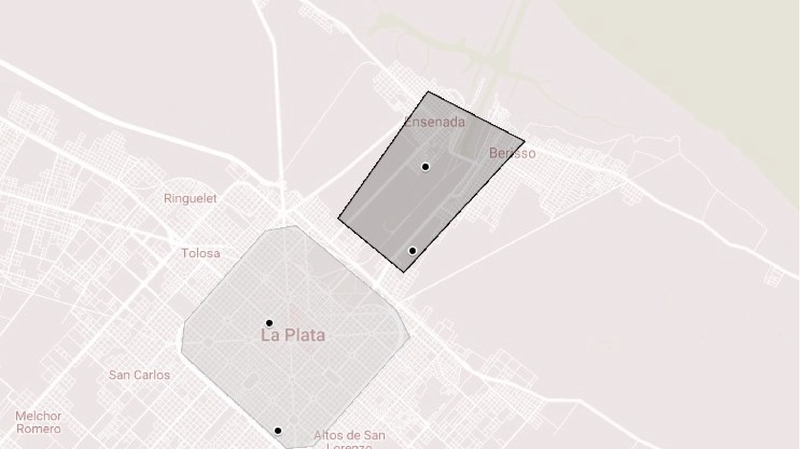
Map of the cities of La Plata, Berisso, and Ensenada, depicting both study areas –industrial zone (dark gray box) and urban zone (light gray box) – and monitoring points (black circles), obtained from Google My Maps.

A total of 78 volunteers between 18 and 62 years old, who lived and worked in La Plata (*n* = 44) and Ensenada (*n* = 34), two regions in Argentina with different pollution levels, were included in this study. La Plata is the capital of the province of Buenos Aires, which has a high vehicle-to-person ratio and was considered the urban zone (U) in this study. Ensenada is a city with high levels of air pollution, mainly due to industrial activity, and was considered the industrial zone (I) [Figure 1]. Both study areas have similar meteorology due to geographical proximity (6 km), with North and North-East winds at an average speed of 15.9 km/h, a humidity of 68.9%, and a temperature of 18.2ºC. The sex distribution of both populations was 45% female and 55% male, and the ages of the subjects from Ensenada and La Plata (mean ± SD) were 34 ± 13 and 29 ± 6 years, respectively. An affidavit of residency and working place was obtained from all volunteers to ensure a minimum of 14 h daily exposure to the study area. Tests were performed simultaneously in both groups in the laboratory of the University Extension Environmental Programme (PAEU, *Programa Ambiental de Extensión Universitaria*) at the Faculty of Exact Sciences (*Facultad de Ciencias Exactas*), the National University of La Plata (UNLP, *Universidad Nacional de La Plata*).

The study was conducted according to the Declaration of Helsinki of 1975, as revised in 2000. The research protocol was approved by the Central Advisory Committee on Bioethics (*Comité Consultivo Central de Bioética*) of the UNLP, and informed consent was obtained from all subjects before they were registered in the study.

The exclusion criteria were as follows: pregnancy, usage of oral/topical antibiotics or prescribed eye medications, or usage of contact lenses. In all participants, medical treatment with drugs of any kind was withheld during the study period.

Previous reports have indicated an expected value of maximal percentage variation expressed as coefficient of variation (CV%) of approximately 60%.^[9,10]^ Based on the results obtained through statistical calculations corresponding to this dispersion value,^[8]^ a total of 30 volunteers per treatment group (zone) were considered adequate to allow statistical conclusions with 80% power (beta, type II error) and a significance level of 0.01 (alpha, type I error).

###  Assessment of Exposure 

PM was utilized as an indicator of air pollution exposure, and samples were collected using a low-volume sampler (PM-2.5 MiniVol${}^{TM}$ TAS; AirMetrics Co., Springfield, Oregon, USA). This draws air at a rate of 5 L/min through an impactor, separating it according to particle size, and a filter, thereby capturing PM.^[15]^ A polytetrafluoroethylene (PTFE) membrane with a 46.2-mm diameter and 2-μm pore-size was used as the filter. Particles of size < 2.5 μm (PM${}_{2.5}$) and aerodynamic diameter ≤ 10 μm (PM${}_{10}$) were both detected. Gravimetric analysis was used to determine the particle content of each sample. Data of PM level in both working areas were obtained through discrete monitoring.

###  Analysis of the Ocular Surface

All analyses were performed in the morning by the same examiner, at the same setting, under the same conditions of temperature and humidity.

###  Questionnaires

The ocular surface disease index (OSDI) and McMonnies (MM) questionnaires in validated Spanish-translated form^[16,17,18]^ were given to all subjects before examination and clinical tests.

###  Ocular Surface Structure

The evaluation of the ocular surface was performed using slit-lamp biomicroscopy with an adapted imaging system (Canon EOS Rebel T3i digital single-lens reflex camera) 30 min after the ST. Aspects of the eyelids, cornea, conjunctiva, and tear film were evaluated and entered into the patient's medical record, according to the Cornea and Contact Lens Research Unit (CCLRU) guidelines and Efron grading scales.^[19,20]^

###  Evaluation of the Tear Film

**Schirmer I test:** Study participants were subjected to the ST (Hub Pharmaceuticals, CA, USA) without the use of topical anesthesia. An obtained value ≤ 10 mm was considered abnormal.^[21]^

**Tear-meniscus Height:** A slit lamp was used to establish the central height of the tear meniscus in relation to the free edge of the lower eyelid. A tear meniscus height < 0.35 mm was considered to indicate low tear volume and suspected dry eye.^[22]^

**Tear Break-Up Time: **TBUT was measured with fluorescein (SF) strips (Hub Pharmaceuticals, CA, USA) moistened with saline solution, which were gently applied to the inferior fornix. A value ≤ 10 sec was considered abnormal.^[23]^

**Meibomian Gland Dysfunction:** Meibomian gland dysfunction (MGD) was observed using digital slit-lamp biomicroscopy^[24]^ and graded using the Efron scale.^[20]^

**Lipid Patterns: **Interferometric images can be acquired using specular reflection techniques. In our study, such images were acquired using a high-intensity slit lamp at a magnification of 25×.^[25]^ The observed patterns were classified according to the system of Guillon.^[26]^

### Corneal and Conjunctival Vital Staining

In this study, vital staining with SF and lissamine green (SLG) was conducted using dye-impregnated strips (Hub Pharmaceuticals, CA, USA) moistened with saline solution that were gently applied to the inferior fornix. The respective pattern of corneal and conjunctival staining was graded,^[19,27]^ and the presence of the lid parallel conjunctival folds (LIPCOF) was taken into account.^[28]^

###  Statistical Analysis

The data acquired were tested for normality and heterogeneity of variance using Chi-Square analysis and Bartlett's test, respectively. Student's *t*-tests were performed for normally distributed data to determine statistically significant differences between two means. A *P*-value < 0.05 was considered to indicate statistical significance. Principal component analysis (PCA) was used for dimension reduction of the data; consequently, the number of variables was decreased to a few principal components (PCs) that accounted for most of the variation. All calculations were performed using Infostat software (Universidad Nacional de Córdoba, Córdoba, Argentina).

##  RESULTS

##  Exposure to Air Pollution

To confirm the previously reported differences in air quality of the two selected areas,^[29,30]^ PM levels at both locations were monitored throughout the study period with the help of local volunteers. Mean values obtained through these discrete measurements throughout the study period are shown in Table 1.

**Table 1:** PM levels in both studied areas are expressed as the annual average. The results are expressed as mean ± SD. The asterisk denotes significant differences (*P* < 0.04) in PM 2.5 levels between populations I and U.


	***n*** ****	***I*** ****	***n*** ****	***U*** ****	***P*** ****
***PM${}_{2.5}$[μg/m${}^{3}$] *** ****	*13*	17.1 ± 8.4*	*7*	10.5 ± 3.6*	*0.024*
**** ***PM${}_{10}$[μg/m${}^{3}$] *** ****	*5*	*41.4 ± 16.4*	*5*	*28.2 ± 9.5*	*0.127*
PM, particulate matter; I, industrial; U, urban; SD, standard deviation; n, number					

The level of PM${}_{2.5}$was significantly higher in zone I than in zone U (P < 0.024, Student's *t*-test), which confirmed the presence of differences in air pollution between the two studied areas.

Moreover, an increase was also detected in the PM${}_{10}$ level in zone I in comparison with zone U. However, the difference was not statistically significant. 

##  Analysis of the Ocular Outer Surface 

Results obtained for the different variables and accompanying parametric comparisons (Student's *t*-test) are shown in Table 2.

**Table 2:** Outer segment characteristics. The values of the number of the sample (*n*) are detailed; the mean (μ); the median (*m*); and the standard deviation (SD) for each study area are provided. The *P*-value is also reported according to the Student's *T*-test. The variables that presented significant differences between populations (*) are indicated in bold font.


**Variables**	**Industrial Zone**						**Urban Zone**			
		**μ**	**** ***m*** ****	**SD**	**μ**	**** ***m*** ****	**SD**	**** ***P*** ****
**Questionnaires**		OSDI score	8.88	4.16	11.8	7.19	4.16	8.68	0.3046
		McMonnies score	7.56	6.50	5.71	7.20	6.00	4.81	0.6749
**Ocular surface**	Without vital staining	Bulbar redness (grade)*	2.79	3.00	0.56	2.39	2.00	0.60	0.0001
		Lid redness (grade)*	2.47	2.50	0,48	2.15	2.00	0.62	0.0009
		Limbal redness (grade)	2.29	2.00	0.78	2.42	2.5	0.71	0.289
		Blepharitis (grade)	0.58	0.00	0.97	0.36	0.00	0.84	0.145
	With vital staining	Fluorescein	Type of cornea (grade)	0.26	0.00	0.65	0.35	0.00	0.62	0.426
		Depth of cornea (grade)	0.18	0.00	0.33	0.27	0.00	0.44	0.220
		Extent of cornea (grade)	0.18	0.00	0.33	0.25	0.00	0.37	0.316
		Conjunctival (grade)*	3.23	3.00	0.73	2.48	3.00	0.64	–
		LIPCOF (grade)	2.24	2.50	0.93	2.42	3.00	0.89	0.231
	Lissamine green	Lissamine green (grade)*	2.17	2.00	1.14	1.25	1.00	0.87	–
**Tear Film**	Volume	Schirmer I Test (mm)*	24.73	29.50	11.20	31.33	35.00	5.39	–
		Tear meniscus height (mm)	0.25	0.25	0.09	0.29	0.30	0.1	0.041
	Stability	TBUT (s)	5.65	5.20	3.12	6.40	5.35	3.46	0.245
	Lipid layer	MGD (grade)*	0.45	0.00	0.63	0.17	0.00	0.36	0.001
	
	
OSDI, ocular surface disease index; LIOCOF, lid parallel conjunctival folds										

With regards to the results shown in Table 2, higher levels of bulbar redness (BR) and lid redness (LR), increased SF and SLG staining values, and an increased MGD grade were noted in the I group compared to the U group. This suggested that the former population were more susceptible to developing higher levels of epithelial damage and MGD than the latter population. In addition, statistically lower mean ST values were obtained in the I group, which indicated abnormalities in the tear film.

No significant differences were present between the groups with respect to the OSDI and MM questionnaires.

**Figure 2:**
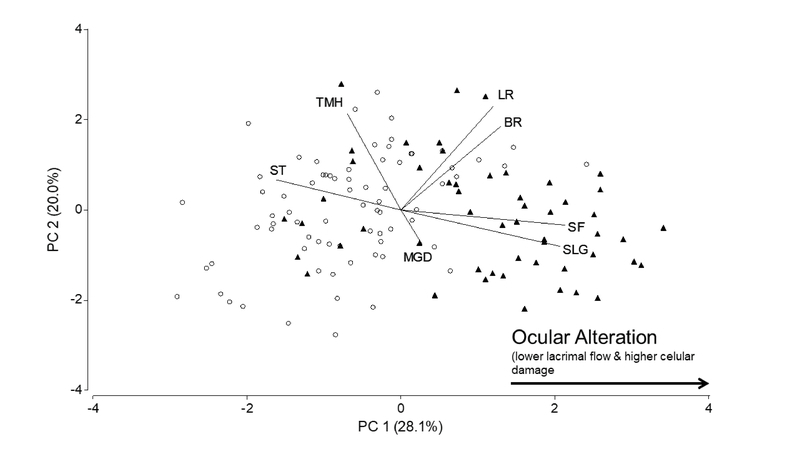
Principal component analysis graph. Each point represents a volunteer: the open circle and black triangle correspond to zones U and I, respectively. The vectors represent the projection of variables: BR, Bulbar redness; LR, Lid redness; SF, Vital staining with fluorescein; SLG, Vital staining with lissamine green; ST, Schirmer I test; and MGD, Meibomian gland dysfunction. Analysis was carried out using the Infostat software.

Consequently, six variables that showed significant differences between the two groups were included in the PCA utilized as a multivariate dimensionality-reduction tool. Data (volunteers in six-dimensional space) were presented as a two-dimensional graph defined by the first two directions of maximal variability of the data points [axis or PC], as shown in Figure 2.

Based on PCA, 54% of the total data variability was attributed to the main plane (PC 1 and 2). The variables of conjunctival staining with SF and SLG were increased to the left on the horizontal axis, indicating a strong positive correlation with PC 1. Conversely, that of ST was increased to the right on the same axis, indicating negative correlation; the two variables had a strong inverse relationship. In contrast, the variables of BR, LR, and MGD were considered to have positive correlations with PC 2. As shown in Figure 2, the areas of provenance of the sample are clearly separated in the horizontal direction of the plot.

##  DISCUSSION

Existing reports in the literature on the effects of air pollution on ocular health are limited to physiological symptoms or signs and/or alterations at a morphological level.^[4]^ As such, reports on the impact of atmospheric pollution on ocular health at a clinical level, which could ultimately progress professional practice, are lacking.

In order to characterize ocular health in individuals exposed to different levels of air pollutants, as well as to identify the associations between effectors and symptoms, reports have indicated the use of various tests and/or questionnaires.^[8,9,11,12]^ The OSDI evaluates ocular alterations based on the symptomatology declared by patients independent of their environmental conditions. In our study, the OSDI was not effective in distinguishing populations exposed to different concentrations of PM [Table 2]. Here, normal values were noted in both populations, in agreement with those reported by Schiffman et al and Miller et al.^[16,31]^ Both the United States National Eye Institute (NEI) and the Tear Film and Ocular Surface Society (TFOS) recommend the use of the OSDI for all optometric/ophthalmological consultations. Nevertheless, objective tests to evaluate the ocular surface should also be performed, since the association between clinical tests and questionnaires used for diagnosis is not adequate.^[32,33]^ Objective clinical tests are essential to identify patients with early alterations who may not present any symptoms.

In our study, significant differences in ocular surface alterations which correlated with increased PM${}_{2.5}$ in the I group were identified [Table 2]. This is consistent with the results of other studies including populations exposed to high air pollution,^[9]^ pollutants related to traffic,^[10]^ and individuals who travel to highly contaminated areas.^[8]^

**Figure 3:**
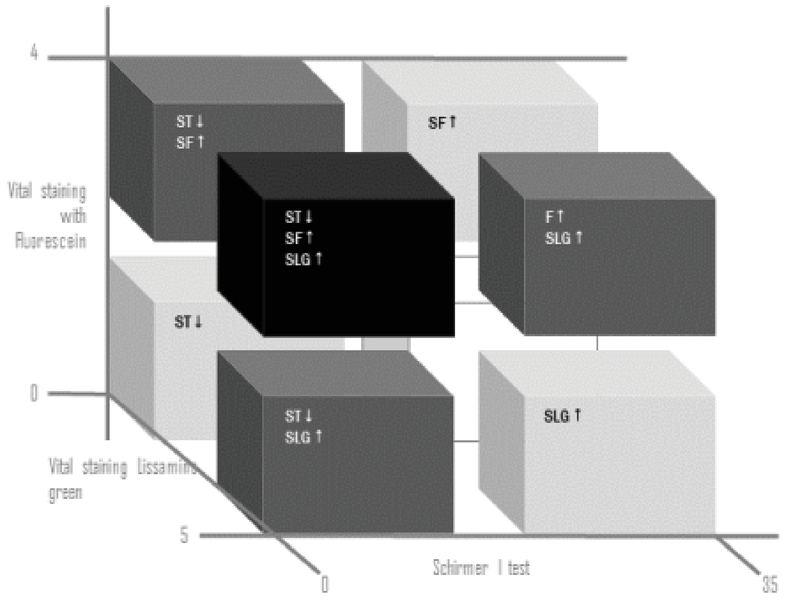
Projection of variables that separate populations with different levels of particulate matter (PM). SF, Vital staining with fluorescein; SLG, Vital staining with lissamine green; and ST, Schirmer test I. The black cube represents the population that exhibits alteration of all three variables, while the white cube represents the population that does not present any alteration of these variables. The cut-off values of each variable were SF: 3; SLG: 3; and ST: 10 mm.

PCA involving the statistically different variables was performed. As a result, ST value and conjunctival SF and SLG staining grades were identified as three variables that could discriminate between individuals from the two zones with different levels of air pollution, indicating their potential use as bioindicators of the health status of the ocular surface. From a clinical point of view, these variables enable classification of the population into eight groups, represented in Figure 3 by eight 3D cubes. From a clinical point of view, these variables enable classification of the population into eight groups, represented in Figure 3 by eight 3D cubes. In this analysis, cut-off values for each variable (SF, 3; SLG, 3; ST, 10 mm) were employed to analyze the effects on the population according to the combination of significant variables against environmental factors.

Regarding the ST axis, the cubes below the cut-off value of 10 mm represent individuals with tear (aqueous) hyposecretion, corresponding to groups with aqueous-deficient dry eye (ADDE);^[4,25]^ the cubes above the cut-off (ST values of > 10 mm) correspond to individuals with clinically normal eyes in terms of basal and reflex tear secretion. Despite noting ST values < 10 mm in some individuals, the mean ST value of both populations was above the cut-off level (I, 24.73 ± 11.2; U, 31.33 ± 0.0033 mm), which indicated that both populations comprised individuals with clinically normal ST values.

The results of our investigation agree with those of Gupta et al who demonstrated a decrease in the mean ST value (22.75 ± 8.91 vs 30.30 ± 7.92 mm) in individuals exposed to high levels of pollutants, and of Saxena who demonstrated lower mean ST values in individuals traveling in heavily polluted areas of New Delhi compared to controls (13.42 ± 6.67 vs 15.95 ± 6.14 mm). The unique finding of our study was that ocular surface cellular alterations, such as an increase in the level of vital staining with both SF and SLG, were correlated with air pollution.

The follow-up of individuals with normal ST values and a trend toward high SF and SLG values is important since tear hyperosmolarity may damage the superficial epithelium by activating inflammatory pathways at the ocular surface, as proposed by Baudouin. Moreover, this cellular damage may cause a loss of goblet cells and dysregulation of the expression of mucins, which leads to instability of the tear film and exacerbation of hyperosmolarity at the ocular surface, thereby reinitiating the dry eye cycle.^[34]^ Consequently, evidence of such ocular surface damage could indicate a high risk of developing recurrent or chronic inflammation in these individuals. Therefore, early detection would enable prompt therapeutic intervention aimed at preventing the development of dry eye disease. Moreover, recent studies have demonstrated PM${}_{2.5}$-induced human corneal epithelial cell damage *in vitro*.^[35]^

Considering the collective findings of previous studies and those of our study, a study to develop a clinical index of ocular alterations including the aforementioned three variables is therefore required. Based on our results, the relationship among these variables (formula) is expressed as the sum of the loads of each variable multiplied by the standardized variable as follows:

$$\begin{array}{cc} \mathrm{𝐹𝑜𝑟𝑚𝑢𝑙𝑎} & =0.7748\times (SF-\mu_{\mathrm{𝑆𝐹}}/{\mathrm{𝑆𝐷}}_{\mathrm{𝑆𝐹}})\\ & +0.7453\times (\mathrm{𝑆𝐿𝐺}-\mu_{\mathrm{𝑆𝐿𝐺}}/{\mathrm{𝑆𝐷}}_{\mathrm{𝑆𝐿𝐺}})\\ & -0.5944\times (\mathrm{𝑆𝑇}-\mu_{\mathrm{𝑆𝑇}}/{\mathrm{𝑆𝐷}}_{\mathrm{𝑆𝑇}}) \end{array}$$

Where ST, SF, and SGL are considered as coefficients. ST, Schirmer I test; SF, vital staining with fluorescein; SGL, vital staining with lissamine green; μ, mean; SD, standard deviation.

Further studies to validate the relationship between air pollution and effects at the ocular level are required to develop tools to facilitate the diagnosis of patients and enable the differentiation of individuals with ocular surface alterations sensitive to different levels of air pollution. Moreover, given the increased prevalence of dry eye worldwide,^[36,37,38,39,30,41]^ the early detection of incipient and asymptomatic alterations is important, since it is a key aspect for improving patients' quality of life.^[42]^

In conclusion, subjects exposed to higher levels of PM in outdoor air presented greater ocular surface alterations. Our study highlights that ST, SF, and SLG values have potential uses as convenient indicators of adverse health effects due to exposure to air pollution.

## Financial Support and Sponsorship

The authors would also like to thank the *Universidad Nacional de La Plata* (UNLP), the *Consejo Nacional de Investigaciones Científicas y Técnicas* (CONICET) and the *Centro de Investigaciones Científicas* (CIC) for their financial support to the present study.

## Conflicts of Interest

There is no conflict of interest.
